# New Jersey State Dental Society

**Published:** 1900-11

**Authors:** William H. Trueman


### NEW JERSEY STATE DENTAL SOCIETY.

The thirtieth annual session of the New Jersey State Dental Society convened at Asbury Park, July 18, 1900. The meeting was called to order by the President, Dr. Wm. E. Truex, of Freehold, shortly after eleven o'clock. His address was a model in its way,—short, well worded, and appropriate to the occasion. He invoked the thankfulness of the society that death had not invaded their ranks during the past year. He announced, as a pleasurable event, the organization of a local society in the southern part of the State. He then, in a few words, referred to the purposes of the Society, and suggested how they could be best attained at the present session. He reminded the members that they owed a duty to themselves, to the State, and to the profession at large. It was the privilege of each to do something for the general good of all as well as to profit by that which others might do. It was their duty to the State, and should be their pride, to keep their State Society well to the fore in the progress the profession was making; and their duty to the profession to see to it that the session added something worth the adding to dental science. As a matter demanding attention, he stated that he had recently been made aware of the fact that the members of the dental profession in New Jersey were not exempted from jury duty. This, he thought, should receive their earnest attention. They were entitled to and should receive as much consideration in this respect as did the medical profession, and he urged that prompt measures should be taken to bring it about. The address was confined closely to local matters, and his suggestions, few and terse, will probably go farther than they would if obscured by a verbose oratorical display.

The afternoon was given up to sight-seeing, the society convening for the evening session at eight o'clock. Dr. H. B. Tileston, of Louisville, Ky., read an interesting paper upon non-cohesive gold. He had, he stated, no new ideas to present upon so trite a subject. His only object in presenting the paper was to keep before the profession an excellent filling-material which seemed to have been, by a large number of dental practitioners, relegated to unmerited oblivion. Its preservative properties, properly used in selected cavities, could not be gainsaid; nor yet could they be scientifically explained. Dr. Miller, it is true, had found that for a limited time non-cohesive gold seemed to have a restraining influence upon germ growth, but so very slight and so soon exhausted was this virtue, it could hardly enter into the question. He attributed its disuse largely to the fact that the modes of instruction almost universally employed in dental colleges made it inexpedient to teach the use of non-cohesive gold in college clinics. While its employment presented no more difficulties than that of cohesive gold, the technique of these forms of gold-foil differed widely. The filling of a cavity with cohesive gold demands the most careful and accurate attention to every minute detail from beginning to end; each particular piece must be placed and immovably held where it is intended to remain, and its condensation effected at once; while the adaptation to the walls of cavities requires a precision of manipulation and an intensity of application on the part of the operator which but few, he believed, were capable of. For these reasons the teaching of the use of cohesive gold afforded splendid manual training for the student, and a cultivation of that precision of technique which is so essential to the successful practice of dentistry. One who has become proficient and skilful in the use of cohesive gold-foil will very readily acquire the methods employed in working non-cohesive, but it is not so easy for one trained to the use of the latter to successfully manipulate the former. He suggested that the use of non-cohesive gold should be taught, and its use in such cavities as especially call for it encouraged in the college clinics during the later part of the term. He then proceeded to describe his methods of using it in various cavities. While nothing especially new was brought out by the paper or its discussion, nevertheless it was well calculated to do what the essayist desired,—to keep before the profession the time-saving, the labor, and tooth-saving qualities of non-cohesive gold-foil.

Dr. F. L. Fosheim, of New York, followed with a paper upon "The Combination of Oxyphosphate Cement with Gold and Amalgam Fillings." In this paper I noted nothing especially new. He advocated the use of cement as a lining to the cavity, usually placing the metallic filling while the cement is still soft, in some cases utilizing its adhesiveness to assist in retaining the filling. The possibility of pulp injury from the presence of the cement was touched upon. Judiciously used, he did not consider this an important factor, and suggested that in many cases of pulp injury reported, supposed to be due to the cement, other and more plausible causes were frequently present.

*Thursday Morning, July 19.*—Eugene Underhill, M.D., of Philadelphia, read a paper entitled "The Eyes and Teeth: Some Concomitant Pathological Changes." The title carried one back a century or two when books with that title were rather common, especially among the Germans. The later clause of the title to the paper gave it a more modern sound. It is, however, by no means a new subject, but was handled by Dr. Underhill on somewhat new lines. His main purpose was to show how the dentist and the oculist, by closely observing symptoms constantly before them, could be helpful to each other and more helpful to their patients. He described many disorders of the teeth that manifest themselves by symptoms which bring the patient to the oculist, and others of the eye that are first manifested by symptoms referred by the patient to dental territory. He explained at some length the why and the wherefore of this, and the importance of both the dentist and the oculist recognizing the true import of these several conditions. He especially impressed its importance in establishing that degree of confidence between patient and oculist or dentist essential to a successful practice, suggesting that, were an oculist consulted, and he, after an examination, pronounced the cause a dental trouble, or a dentist consulted, and, at once recognizing the true condition of affairs, referred the patient to an oculist; in either case, the diagnosis proving correct, the confidence, the reputation thus acquired in a single instance might lay the foundation for a large and lucrative practice. He described at some length symptoms of supposed eye trouble of dental origin; of supposed dental trouble originating within the oculist's domain; and others again closely simulating one or the other, sometimes both, which should be referred to the physician. He laid especial stress upon the more pronounced differentiating signs suggesting the real origin of the trouble; and urged that by close observation, with these as a guide, the more subtile, but yet more convincing evidences each must learn for himself be studied and thoroughly mastered. An important part of his paper was that devoted to the early diagnosis of malignant growths and the diagnostic signs of syphilis seen about the eyes. He urged that the importance of this alone was sufficient incentive for a dentist to be, so far as knowing and being keenly alive to the meaning of these signs which are so prominently and constantly before him are concerned, somewhat of an oculist; and on the same ground, he urged, it was equally important that an oculist should be somewhat of a dentist; and both sufficiently conversant with general medicine to distinguish a trouble expressed at its seat from one reflected, and to know whether it required for its relief a dentist, an oculist, or a general practitioner.

It was an excellent paper, one well worthy a careful study when it shall be published in full. Its delivery was marred by the speaker so frequently stopping to inject explanatory remarks, puns, or humorous suggestions. Within bounds, this is, of course, allowable; it is, indeed, when tactfully done, useful to emphasize or clinch an idea. When, however, these breaks occur frequently, as it did in this case, and at times inappropriate, it is undignified; it breaks the thread of the discourse and distracts the attention of the audience.

This was followed by what may be termed a lecture, by Thomas C. Stellwagon, Jr., D.D.S., of Philadelphia, upon "The Blood." It was extempore; it would have been far more impressive had it first been committed to writing and read. The hesitancy, now and again going back to take up a forgotten thought, or to add an overlooked explanation, not only wastes time, but tends to the listeners losing the connection of ideas. He illustrated his remarks by several diagrams, and had in addition a number of microscopic slides conveniently arranged under microscopes. To a large audience, especially when the opportunity is given at the close of a session protracted beyond their usual dining hour, the attempt to show specimens under a microscope requiring delicate adjustment by each observer will always prove a failure. Now that the microscopic field can be so accurately photographed, and by the same art duplicated, or shown upon a screen visible to all at the same time, the instrument itself may well be relegated to the laboratory. It has ceased to be the novelty it once was, and its revelations no longer require for their delineation an artist's pencil, with its coarseness and its unavoidable personal equation.

The doctor has made a special study of the blood, and his remarks were, no doubt, a surprise to many. We learned that red and white corpuscles were not the only bodies floating in this important fluid, nor were they the simple things they were supposed to be in our school-boy days. We learned from him also that modern science had in this, as in other lines of investigation, by its increasingly accurate and far-reaching methods of research, dispelled many illusions of the past. He stated, at some length, the more recent theories regarding the origin of the various bodies found in the blood; their significance, and the diagnostic value of the changes in relative number, size, and form of those usually there found, and the pathological significance of unusual bodies, or those only occasionally met with. He dwelt especially on the importance of these matters in the early recognition of impending or established pathological conditions; at times these may be noted early enough, he stated, to abort or control lesions that become very serious before being otherwise manifest. He spoke also of the *rôle* the blood played in some forms of germ-caused disorders; and of its relation in various ways to germ pathology and germ therapeutics. To one not thoroughly conversant with the subject it is exceedingly difficult to make a satisfactory digest or *résumé* of a lecture like this. It is well worth looking up, when it shall be published, for its value as an *exposé* of up-to-date ideas upon an important subject.

This was followed by an interesting event. Some months ago, as the readers of this journal will no doubt remember, several members of the New Jersey State Dental Society, who were also members of the Dental Protective Association, compounded with the International Tooth Crown Company for infringements, or alleged infringements, of their patents. This led to a much-to-be-regretted acrimonious ventilation of opinions in the dental journals, during which the motives of the gentlemen who compounded, and Dr. Crouse's management of and fidelity to the Protective Association, were called in question. Those who personally knew the gentlemen, and also knew Dr. Crouse, felt very sure that, notwithstanding both sides seemingly had grounds for complaint, it was a matter that would speedily change its complexion when properly understood. Dr. Crouse was present at the meeting the evening before, and asked that opportunity be afforded to clear the matter up. The time appointed was the close of this morning's session. When the time arrived Dr. Crouse took the floor. He gave a detailed account of those actions of his which had been called in question, and in an explanatory mood took up one by one the aspersions which had been cast upon his motives and his management of the Protective Association, especially those which had been assigned as excuses for defection from the ranks of the Protective Association. He asked to be questioned upon any points he had not made clear, and promptly answered all questions put to him. Having given his side of the story, he asked that the others, who were present, would give theirs, that they might, while they were together, face to face, clear the matter up. The applause, so prompt and so generous, was sufficient evidence that the audience accepted and were satisfied with the story he told. He was followed by those who had been accused of being unfaithful to the Protective Association. I see no good reason for repeating the stories told; there are circumstances under which men feel impelled in their own defence to lay bare to their fellows in some measure their private affairs; when they have so done and made good their cause, is it not sufficient to publish this result alone? There was, in each case, special or personal reasons which, at the time they made their settlement with the Tooth Crown Company, left them but little choice, and made that course the wisest for them to follow. Their explanation was quite as satisfactory as was that of Dr. Crouse. The incident may be considered closed; it was—as the editor of the INTERNATIONAL DENTAL JOURNAL suggested at the time, when commenting upon it, it would probably prove—a misunderstanding and nothing more. A little calm correspondence, and a little less haste in rushing into print, would have saved all the unpleasantness and the humiliation of placing on record, for future generations to read, another unethical chapter in our periodical literature that can *never be blotted out.*

The afternoon was set apart for clinics. Dr. W. A. Capon, of Philadelphia, illustrated a method of anchoring porcelain sections (inlays) with platinum wire, insuring thereby a more secure hold in the cement. He was surrounded all the afternoon by a crowd of attentive observers.

Dr. George Evans, of New York, demonstrated, with specimens, a method of facing metallic shell crowns with porcelain,—a method he has published, and which is perhaps well known. I was not impressed with its value. The porcelain facing is necessarily frail, and upon bicuspid teeth has no protection from the stress of occlusion. In such cases the inlay method has decided advantages. A very small surface of porcelain is all that is needed, and that can be so placed as to be perfectly protected. The edges of the porcelain in the specimens Dr. Evans exhibited were very thin, and did not present a smooth and even outline.

The other clinics were of minor importance.

The exhibitors made a very creditable display. This has been of late years made a notable feature of the New Jersey State Society meetings, and an instructive one. The large room of the Auditorium is an ideal place for the purpose. It is sufficiently large to accommodate all, and as they are spread out, side by side, ample opportunity is afforded to see all they have to show, and to compare similar manufactures, one with another. This has tended, I am impressed, to restrain to a great extent the trade features.

The attendants are there more as exhibitors than as salesmen; they seem more anxious to show their goods than to solicit customers. It is impossible, with the space at command, to even name the firms represented, much less to describe their exhibits. The Weber fountain spittoon, with a porcelain bowl, for neatness and cleanliness, deserves especial mention. An electric throat and mouth light, made by the Unique Electric Device Company, of New York, costing, complete, with battery, five dollars and fifty cents, is the most compact arrangement I have yet seen. The light was very bright, with less than the usual heat. William Denker, of Dorchester, Mass., exhibited an outfit for quickly forming cavities in porcelain teeth, costing one dollar and fifty cents. It consisted of suitable tools fitting to the dental engine hand-piece, and an abrasive paste. It seemed a practical idea.

The Hammond electric furnace and rheostat, for crown-, bridge-, and inlay work, and an electric dental engine by the Ritter Dental Manufacturing Company, designed for an alternating current, having all the advantages of those made for direct current, are novelties worthy of mention.

At the evening session Dr. J. Allen Osmun, of Newark, N. J., read a paper entitled "Dental Jurisprudence in its Relation to State Examining Boards; the Profession; the Laity."

This was a well-written paper upon an important phase of the subject, the doctor's attention having been directed, by reading a series of articles in a medical periodical, to a line of argument contesting the right of State Legislatures to curtail the rights and privileges of practitioners of medicine and dentistry by making the exercise of that right dependent upon the permission of so-called examining boards. He formulated a series of questions covering the contested ground, and submitted them to counsel, asking not simply their opinion, but also the grounds or authorities upon which their opinions were based, and for such other information as they might be able to give pertaining to the subject. This information was paid for, and was as carefully prepared as it would have been to meet a case at court involving the points at issue. It having been contended that the exercise of the power lodged in examining boards conflicted with the Constitution of the United States, especial attention was therefore first given to this point. His paper was largely made up of quotations from opinions thus obtained, of authorities cited, and of such extracts from current periodicals as he thought would make clear the points he wished to present. He simply made himself the mouth-piece of those consulted, learned in legal matters and having no bias one way or the other to swerve their judgment. This gives to the paper an importance and a claim on professional attention to which very few of the many dissertations on this and allied subjects have been entitled.

The questions were, first, Has the State the power to prescribe the qualifications and conditions upon which a person shall be admitted or licensed to practise the profession of dentistry?

Second, Has the State the power to regulate and control the practice of this profession after a person has once been admitted or licensed to so practise?

He assumed that dentistry was one of the skilled and learned professions, standing on the same basis as law or medicine; and that, whatever power the State has over the matters referred to is derived from what is called the "police power." This, in its broadest acceptation, means the general power of a government to guard and preserve the public welfare, if need be, at the expense of private rights. It is one of the inherent powers of government, and, like the power of eminent domain, exists independent of, and is rather limited than created by the Constitution. It is necessarily despotic in its character, and individual and property rights, beyond the express constitutional limits, must yield to its exercise. The doctor, at some length, noted the various ways in which this power was exercised without question, all of which were as arbitrary and as much an invasion of private and property rights as is its application to dental or medical practice. Among these were the regulation of the speed of railroad trains and of trolley cars, preventing the adulteration of food, restricting the sale of dangerous drugs or intoxicating liquors. This police power, he said, can only be exercised by legislative enactment, and it rests solely within legislative discretion to determine when the public welfare or safety requires its exercise. It is an established principle in this country, that so long as the Legislature does not pass the limits fixed by the Constitution the courts have no authority to interfere, on the ground that the legislative acts in question violate a natural principle of justice and right. He then quoted a number of decisions sustaining these positions, and also disposing of the argument that the Constitution of the United States conferred certain rights and privileges which the dental and medical laws unduly restricted. He contended that the evidence collected and presented placed beyond question the right of State Legislatures to determine who should and who should not practise either profession, the right to determine and to fix the qualification necessary, to establish such means, and to appoint such agents as they may deem requisite to ascertain whether or not the applicant possesses the knowledge and skill a safe and efficient exercise of the desired privilege calls for. The right to change from time to time these requirements was equally unquestionable; as was also the right of each State to maintain its own standard, and to insist on ascertaining for itself, in its own way, without regard to what any other has done or might do, the qualification of each and every applicant. The acceptance by one State as satisfactory of the standard and examinations of another is merely a courtesy extended; it cannot be claimed as a right. The constitutional provision that the citizens of each State shall be entitled to all the privileges and immunities of citizens in the several States does not mean that a citizen of the State of Indiana who comes to the State of New Jersey brings with him into this State the privileges and immunities which he enjoyed as a citizen of the former State, but rather, that upon his arrival here in this State he at once becomes possessed of all the privileges and immunities which pertain to citizenship of this State. In support of this view the doctor referred to the ease and readiness with which a divorce could be obtained, for instance, in South Dakota. A citizen of that place coming to reside in New Jersey could not demand from the New Jersey courts a like quick and easy divorce; he would be compelled to abide by the requirements of New Jersey law as fully as though he had always resided within the State.

He next referred to the preliminary education clause of the New Jersey Dental Law. It calls for a preliminary education equal to that furnished by the common schools of this State. This has been held to mean a full High School course, an education equal to that ordinarily provided by the High Schools of the State. This, he is inclined to think, is an error. The Constitution of the State requires the Legislature to provide for the maintenance and support of a thorough and efficient system of free public schools for the instruction of all children between the ages of five and eighteen years. It is manifestly impossible to provide for every child in the State a High School education; to attempt to do so would at once break down the entire system. The wealthier districts and the larger cities have these schools as part of their peculiar advantages, not as constitutional requirements, but rather through the exercise by the Legislature of a power therein existing beyond the constitutional obligation. He therefore thought, and quoted judicial decisions in support of this, that the educational requirement of the dental law was one equal to that of the common schools, and that graduation from a High School is not a necessary qualification to entitle a person to an examination for a license to practise dentistry in New Jersey. This brief *résumé* merely gives the gist of his paper; it must be read in its entirety to fully understand its purport and to appreciate its careful preparation.

Dr. G. Carlton Brown, of Elizabeth, N. J., who led in the discussion, questioned very much Dr. Osmun's suggestion that a common school education, as that term is used in the dental law, did not refer to a High School. He stated that it was the understanding of those who framed the law that it called for a qualification equal to that of a graduate of a four-year High School course, and was so understood by the Superintendent of Public Instruction. Referring to the New Jersey State Board of Examination resigning from the National Board, he said he did not see how they could do otherwise and still maintain their present high standard. It was understood that all State boards having membership in the national body accepted without question the colleges they recommended, many of which were contented with a preliminary education far below that required by the New Jersey board. To continue membership and refuse to recognize the standard adopted by the national board was not consistent with his ideas of honor; to lower the standard of New Jersey would be a step backward, would be to lose much that they had been so long striving for, and inadvisable. The discussion was an earnest one, mainly centring upon local matters. To many the resignation from the National Board of Dental Examiners, notwithstanding Dr. Brown's statement, was thought to be a disadvantage to the State, and later, a resolution was adopted asking the State board to withdraw their resignation, it being understood that it had not been as yet accepted by the national body, and to continue to work with them, hoping that by so doing they might be instrumental in inducing other State boards to, as soon as practicable, bring their standards to a uniform requirement of a full High School course.

*Friday Morning.*—Dr. Meeker read an interesting sketch of his twenty-five years' stewardship as secretary of the Society. He has been an earnest and faithful officer, and much of the success of the Society and its more notable features are due to his tireless efforts in its behalf.

The following officers were elected for the coming year: President, Dr. F. Edsall Riley, of Newark; Vice-President, Dr. Wm. L. Fish, of Newark; Secretary, Dr. Chas. A. Meeker, of Newark; Treasurer, Dr. Henry A. Hill, of New Brunswick.

William H. Trueman.

